# Recent Speciation in Three Closely Related Sympatric Specialists: Inferences Using Multi-Locus Sequence, Post-Mating Isolation and Endosymbiont Data

**DOI:** 10.1371/journal.pone.0027834

**Published:** 2011-11-15

**Authors:** Huai-Jun Xue, Wen-Zhu Li, Rui-E Nie, Xing-Ke Yang

**Affiliations:** 1 Key Laboratory of Zoological Systematics and Evolution, Institute of Zoology, Chinese Academy of Sciences, Beijing, China; 2 Graduate University of Chinese Academy of Sciences, Beijing, China; American Museum of Natural History, United States of America

## Abstract

Shifting between unrelated host plants is relatively rare for phytophagous insects, and distinct host specificity may play crucial roles in reproductive isolation. However, the isolation status and the relationship between parental divergence and post-mating isolation among closely related sympatric specialists are still poorly understood. Here, multi-locus sequence were used to estimate the relationship among three host plant–specific closely related flea beetles, *Altica cirsicola*, *A. fragariae* and *A. viridicyanea* (abbreviated as AC, AF and AV respectively). The tree topologies were inconsistent using different gene or different combinations of gene fragments. The relationship of AF+(AC+AV) was supported, however, by both gene tree and species tree based on concatenated data. Post-mating reproductive data on the results of crossing these three species are best interpreted in the light of a well established phylogeny. Nuclear-induced but not *Wolbachia*-induced unidirectional cytoplasmic incompatibility, which was detected in AC-AF and AF-AV but not in AC-AV, may also suggest more close genetic affinity between AC and AV. Prevalence of *Wolbachia* in these three beetles, and the endosymbiont in most individuals of AV and AC sharing a same *wsp* haplotype may give another evidence of AF+(AC+AV). Our study also suggested that these three flea beetles diverged in a relative short time (0.94 My), which may be the result of shifting between unrelated host plants and distinct host specificity. Incomplete post-mating isolation while almost complete lineage sorting indicated that effective pre-mating isolation among these three species should have evolved.

## Introduction

In small herbivorous invertebrates, major changes in host taxa preferences are generally thought relatively rare [1] with less than 20% of speciation events accompanied by a shift to a different plant family [2], [3]. Closely related species most often use closely related hosts, presumably because of the ease of adaptation to hosts already used by a lineage of herbivores. What circumstances would initiate shifts to distantly related host? When incipient herbivore species are sympatric, host-related selection and reproductive isolation become interdependent, which may favor adaptive feeding on more distantly related hosts. Studying the relationships between parental divergence and post-mating isolation among closely related species in sympatry, therefore, will provide insights into natural selection pressures, i.e. reinforcement, which favor feeding adaptation and ecological speciation. Indeed, in some cases, host specificity alone can act as an almost complete pre-mating barrier among insect populations [4], [5]. Therefore, major host shifts may be of fundamental relevance to mechanisms of ecological speciation. However, the relationship between parental divergence and post-mating isolation among closely related sympatric specialists are still little understood though they may be useful as indicators of sources of natural selection (e.g., reinforcement) related to speciation [6]–[8].

*Altica* Geoff. (Insecta: Coleoptera: Chrysomelidae) is a species-rich genus of flea beetles of which more than 300 species have been described [9]. The *Altica*-host plant system has been suggested as a candidate for ecological speciation studies because many species in this beetle genus are specialists and some closely related species are sympatric, suggesting the possibility that some speciation events may be associated with host switching [10], [11]. To our knowledge, more than 30-family plants were colonised by about 70 *Altica* species whose host plants were recorded, furthermore, most of these beetles are specialists [12]. Because host plant switches between families are unusually frequent in *Altica* species, the possibility of a strong source of selection for ecological divergence is raised.

Among them, *Altica fragariae* Nakane, *A. viridicyanea* (Baly) and *A. cirsicola* Ohno (abbreviated as AC, AF and AV respectively) are distributed sympatrically over much of East Asia, and even co-occur in some microhabitats. Preliminary molecular phylogenetic analyses, with incomplete sampling of species and based on single individuals of each species using mitochondrial DNA sequence data, nevertheless indicated that these three species are very closely-related and may form a monophyletic group [13]. These analyses also suggest that AF is the sister to “AV+AC” [13]. Previous studies also have shown that hybrids between AF and AV can be obtained under laboratory conditions [10], [14], further indicating their close genetic affinity. Although the possibility remains that there are other, allopatric species more closely related to each of our three study species here, the incomplete reproductive isolation of these sympatric species using distinct hosts requires explanation. The purpose of this study is twofold. First is to use phylogeny analyses of multiply DNA markers to characterize the nature and extend of reproductive isolation among the above three species. Second is to infer the possible sources of natural selection.

*Wolbachia* is maternally inherited intracellular bacteria that infect a wide range of arthropods and nematodes [15], [16]. Many studies have suggested that crosses of *Wolbachia*-infected males and uninfected females (or females infected with a different incompatible strain) are cytoplasmically incompatible and results in high levels of embryonic mortality among the offspring [15], [17]–[20]. In our study system, unidirectional incompatibilities were found in AC-AF and AV-AF combinations [10] (and see the results of present study), that may give a typical example of *Wolbachia*-induced cytoplasmic incompatibility (CI) between closely related species. In fact, the essence of the post-mating reproductive isolation (*Wolbachia*-induced or nuclear-induced incompatibilities) is important for us to understand the process of beetle speciation. Furthermore, the sequence data of bacterial endosymbiont, *Wolbachia* may also provide phylogenetic evidence of the hosts because of its vertical transmission [21]–[23], although horizontal transmission in many cases was reported [24]–[26].

The present study is designed to test two alternative hypotheses: (1) AF and AV are more closely related to each other than either is to AC; and, (2) AV and AC are sister species which demonstrate incomplete post-mating isolation. Accordingly, we have re-estimated the species relationship using multiple loci and more individuals, re-evaluated the pairwise post-mating reproductive isolation among the three species, detected the infection status of beetles by *Wolbachia* and analysed the relationship of *Wolbachia* among these three beetles in present study. Furthermore, we estimated the relative timing of speciation of these species based on mitochondrial data. Our hope is to contribute to a better understanding of the mechanism and the process of (ecological) speciation of specialists via host plant shifting to distantly related hosts.

## Materials and Methods

### Samples

Six individuals of AC from two locations, nine individuals of AF from four locations and five individuals of AV from three locations were collected for beetle phylogenetic study, and one individual of *A. koreana* Ogloblin was selected as the outgroup (Table 1). Total 127 individuals of beetles (23 AC from two locations, 47 AF from four locations, and 57 AV from four locations) were collected for bacterial endosymbiont study (Table 2).

**Table 1:** List of specimens and collecting data for beetle phylogenetic study.

Species	Location	Geographical coordinates	Sample size	Sampling date
AC	Shahe	40.165′N, 116.217′E	3	2009.VII.14
	Botanical Garden	40.001′N, 116.206′E	3	2009.IX.21
AF	Badaling Forestry Centre	40.341′N, 116.008′E	2	2009.VII.14
	Taotiaogou	40.635′N, 116.537′E	2	2009.IX.25
	Beikouzi	40.539′N, 116.417′E	4	2009.IX.26
	Badaling	40.332′N, 116.035′E	1	2010.V.12
AV	Badaling Forestry Centre	40.341′N, 116.008′E	2	2009.VII.14
	Taotiaogou	40.635′N, 116.537′E	2	2009.IX.25
	Xingshou	40.298′N, 116.335′E	1	2009.IX.26
AK	Ming Tombs	40.2′N, 116.2′E	1	2004.IX.10

**Table 2:** List of specimens and collecting data for bacterial endosymbiont study.

Species	Location	Geographical coordinates	Sample size	Sampling date
AC	Shahe	40.165′N, 116.217′E	19	2009.VII.14, 2010.V.12
	Beishatan	40.004′N, 116.381′E	4	2009.VII.25
AF	Matao	40.142′N, 115.787′E	16	2010.IX.9, 2010.IX.15
	Taotiaogou	40.635′N, 116.537′E	15	2009.IX.25
	Beikouzi	40.539′N, 116.417′E	12	2009.IX.26, 2010.VI.10
	Sijiasui	40.091′N, 115.948′E	4	2010.IX.2
AV	Matao	40.142′N, 115.787′E	15	2010.IX.9, 2010.IX.15
	Taotiaogou	40.635′N, 116.537′E	19	2009.IX.25
	Beikouzi	40.539′N, 116.417′E	14	2009.IX.26, 2010.VI.10
	Sijiasui	40.091′N, 115.948′E	9	2010.IX.2

### DNA extraction, amplification, and sequencing

Total genomic DNA was extracted from whole beetles by puncturing and soaking the specimens in extraction buffer overnight [27] using the TIANamp Genomic DNA Kit (Tiangen, Shanghai, China). After extraction, beetle specimens were retained as vouchers deposited in the Institute of Zoology, Chinese Academy of Sciences, Beijing, China.

DNA sequences from the nuclear and mitochondrial genomes of each species were used in this study. The four sequenced gene regions were: (1) a fragment of mitochondrial cytochrome oxidase 1 gene (COI 801 bp); (2) a fragment of mitochondrial cytochrome oxidase 2 gene (COII 508 bp); (3) the complete second internal transcribed spacer of the nuclear ribosomal RNA cluster (ITS-2∼350 bp); and (4) a fragment of nuclear protein-coding gene elongation factor 1-alpha (EF1α: ∼695 bp exon and ∼525 bp intron). Standard PCR protocols were used to amplify COI and COII using two overlapping PCR amplifications [13], [28], PCR amplification included: a pre-cycle denaturation at 94°C for 5 min, a post-cycle extension at 72°C for 8 min, and 30 cycles of a standard three-step PCR (94°C for 1 min, 50°C for 1 min, 72°C for 1 min); ITS2 was amplified using the primers anchored in the 5.8S and 28S rDNA region [29], [30]: a pre-cycle denaturation at 94°C for 2 min, a post-cycle extension at 72°C for 2 min, and 35 cycles of a standard three-step PCR (94°C for 1 min, 57°C for 1 min, 72°C for 1 min); EF1α was amplified with two primer sets [31] using a modified touchdown PCR protocol: 94°C for 4 min, 19 cycles at 94°C for 30 s, decreasing the annealing temperature from 62°C to 43°C for 1 min, 72°C for 1 min, then 26 cycles at 94°C for 30 s, 42°C for 1 min, 72°C for 1 min, and 72°C for 7 min as a final extension. All the primers were listed in Table 3.

**Table 3:** Sequence of primers used to amplify the gene fragments.

Locus	Primers name	Sequence of primers
COI+Leu+COII	Jerry	CAACATTTATTTTGATTTTTTGG
	Pat	TCAATTGCACTAATCTGCCATATTA
	P1	GACTTCAATTTAACCCACCA
	Barbara	CCACAAATTTCTGAACATTGACCA
ITS2	FB5.8SFWD	CTGGACCACTCCTGGCT
	FB28SREV	GGTAGTCTCACCTGCTCTG
EF1- a	EFS149	GARAARGARGCNCARGARATGGG
	EFA1106	GTATATCCATTGGAAATTTGACCNGGRTGRTT
	EF1α-SN	TGGGAAAAGGYYCCTTCAAATATGC
	EF1α-AN	CRTRACCACGACGYAATTCTTTGACAG
*wsp*	*wsp*81F	TGGTCCAATAAGTGATGAAGAAAC
	*wsp*691R	AAAAATTAAACGCTACTCCA

PCR products were purified using the QIAquick PCR clean-up kit (QIAGEN), and a Perkin-Elmer BigDye terminator reaction protocol was followed to generate sequences in a PerkinElmer ABI3700 automated sequencer using the same primers for amplification reactions. PCR products were sequenced directly using the same primers as above and the BigDye Terminator Cycle Sequencing kit (Applied Biosystems, Foster City, CA, USA). All of the fragments were sequenced in both directions.

The sequence files were aligned and edited using CodonCode Aligner 2.02 (CodonCode, Dedham, MA, USA). Variable sites were identified using the automated mutation detection function in CodonCode Aligner, followed by manual inspection of electropherograms and additional resequencing of ambiguous bases. Successful PCR amplification and sequencing of target genes were confirmed by aligning the resulting sequences to other closely related species in the Chrysomelidae, verifying the correct reading frame without stop codons. All Sequences are deposited in GenBank under the accession numbers JN903042–JN903103 (COI/COII: JN903042–JN903061; EF1α: JN903062–JN903082; ITS2: JN903083–JN903103).

### Gene tree estimation

All EF1α and ITS2 sequences appeared homozygous, as judged on the basis of a lack of double peaks in chromatograms from both directions. The nuclear gene, EF1α was partitioned into exon and intron to allow for variable evolutionary rates between gene regions.

Gene trees were estimated with the following seven combinations: COI, COII, COI/COII (along with the intervening leucine tRNA, COI+Leu+COII), EF1α exon, EF1α intron, EF1α (exon+intron), ITS2 and concatenated data (five partitions: COI, COII, EF1α exon, EF1α intron and ITS2). The best-fit model of nucleotide substitution for each partition or each combination was selected using the Akaike Information Criterion (AIC) in jMODELTEST 0.1.1, a program recommended to supercede ModelTest [32]. Congruence among partitions was assessed by the ILD test [33], [34] implemented in PAUP* 4.0 [35].

Both maximum parsimony (MP) and maximum likelihood (ML) analysis were performed with PAUP* 4.0. For MP tree construction, one hundred replicates of a heuristic search were performed with an initial random stepwise addition of sequences and with TBR branch-swapping. Branch support was estimated from 1000 replicates of a bootstrap search. ML analyses were performed with a heuristic search, stepwise addition, 100 replications and TBR swapping. Support was measured with 100 bootstrap replicates. Bayesian analyses were carried out with the program MRBAYES version 3.1 [36], [37]. The settings were two simultaneous runs (each with two Markov chains) of the Markov chain Monte Carlo (MCMC) for 2×10^6^ generations, sampling every 100 generations. The first 25% generations were discarded as the burn-in. Log likelihood plots of trees from the Markov chain samples were examined in TRACER version 1.5 [38] to determine convergence to a stable log likelihood value. BMCMC posterior probability (PP) values represent the proportion of MCMC samples that contain a particular node. Bayesian analyses on the combined data were performed with a mixed model, estimating model parameters separately for each data partition (COI, COII, EF1α exon, EF1α intron and ITS2).

### Species tree and divergence time estimation using multiple-allele DNA sequence data

**Figure 1:**
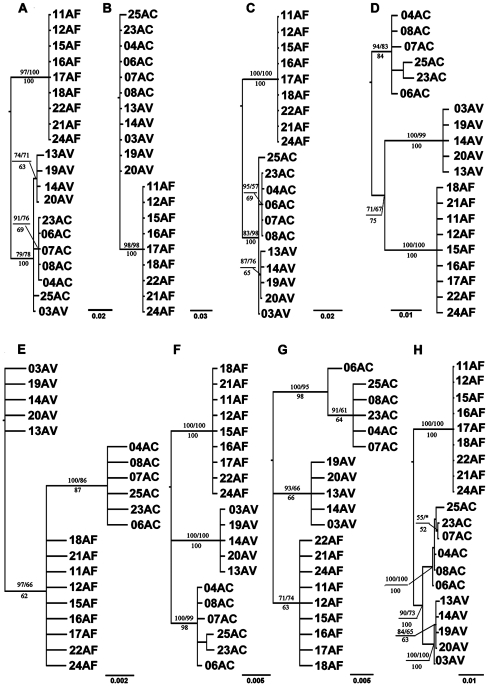
Phylogenetic cladogram of the *Altica* based on different gene combines. The values above the branches represent Bayesian posterior probabilities and Maximum Likelihood bootstrap support values, respectively. The values below the branches represent Maximum Parsimony bootstrap support values. The outgroup AK was moved away. Nodes with ≥50% bootstrap value or posterior probability are labelled. a. based on COI, b. based on COII, c. based on COI/COII, d. based on intron of EF1α, e. based on exon of EF1α, f. based on whole EF1α, g. based on ITS2, h. based on concatenated data.

Because the above gene trees using single gene partition are discordant (see result, Fig. 1), we tried the program *BEAST, which is a part of the BEAST v1.6.1 package [39] to estimate species tree. *BEAST can do bayesian estimation of species trees from multilocus data under the coalescent model, an extension of the coalescent prior designed to handle multiple species, and also can handle different numbers of gene copies for each taxon. Furthermore, *BEAST infers species trees from multilocus data and shows advantages in computational speed and accuracy over other similar methods [40], [41]. All of the five partitions were employed for the species-tree analysis.

The program *BEAST also allow for joint estimation of the species tree and divergence times [39]. And a recent study showed that divergence time estimated from the multilocus species tree are more precise than that with gene-tree based approaches [40]. Due to the lack of fossils for *Altica* beetles, direct calibration of the tree topologies was difficult. Instead, branch lengths and node ages in the BEAST analysis were estimated by applying a gene-specific substitution rate. In present analysis, only mtDNA sequence data were used with average pairwise divergence rates as 1.73% My^−1^ for COI and 1.38% My^−1^ for COII [41]. A relaxed clock with log-normal branch length distribution was used and a Yule speciation model was applied to model population size through time, other prior parameters were set as default.

### Post-mating reproductive isolation

Over-wintered adults were collected from field populations living on their host plants in the early summer. To avoid potential interspecific hybrid individuals, allopatric populations (only one *Altica* species was detected at each site) were selected: *A. fragariae* at Badaling, Yanqing (40.332°N, 116.035°E), *A. viridicyanea* at Liucun, Changping (40.110°N, 115.995°E), and *A. cirsicola* at Shahe, Changping (40.165°N, 116.218°E) of Beijing, China. In the laboratory, cultures of these insects were maintained in their native host plants in growth chambers at 16∶8 LD and 25°C. *Duchesnea indica* (Andrews) (Rosaceae) (the primary host plant of *A. fragariae*), *Geranium nepalens* (Sweet) (Geraniaceae) (the exclusive host plant of *A. viridicyanea*) and *Cirsium setosum* (Willd.) MB. (Asteraceae) (host plant of *A. cirsicola*) were locally collected, kept refrigerated, and used within one week.

To determine the viability of eggs arising from each cross, virgin adults from each cross were sexed, then five pairs (either intra- or inter-specific) were put in each glass jar (11.5 cm in height and 12.0 cm in diameter) for mating experiments (Table 4). Five to ten replicates were conducted for each. Usually, egg collection started from the 10th day after eclosion to make sure the beetles sex mature and the mating occurred for each replication. The eggs were collected and branches were replaced for five times every other day. The eggs were gently scraped and put in 9-cm Petri dishes lined with moistened filter papers and the newly hatched larvae were checked daily.

**Table 4:** Hatch percentage of each cross among three *Altica* species, AC, AF and AV.

Cross (n)	Hatch percentage (±SD) of eggs
AC♀×AC♂ (6)	92.23±3.49 a
AF♀×AF♂ (5)	89.83±4.16 a
AV♀×AV♂ (6)	89.19±3.92 a
AC♀×AF♂ (10)	49.12±11.61 bc
AF♀×AC♂ (10)	6.44±4.95 e
AC♀×AV♂ (10)	28.86±34.20 d
AV♀×AC♂ (10)	42.15±21.73 cd
AF♀×AV♂ (5)	1.19±2.66 e
AV♀×AF♂ (5)	65.79±16.17 b

Upon hatching, the larvae were split into two or three cohorts and placed in Petri dishes of 9-cm diameter containing a moistened filter paper and fresh leaf material (the combines are showed in Table 5). About 20 (17–29) larvae were introduced in each Petri dish, and 4–15 replications were run for each cross (sample sizes for each cross are given in Table 5). Fresh leaves were added as needed, and mortality was checked daily until the larva died or reached the third instar.

**Table 5:** Survival percentage (±SD) to the 3^rd^ instar of each cross on the specific host plant of AC, AF and AV respectively.

Cross	Survival percentage on different plants		
	*Cirsium setosum*	*Duchesnea indica*	*Geranium nepalens*
AC♀×AC♂	90.04±5.60 (13)	0 (5)	0 (5)
AF♀×AF♂	0 (5)	89.87±8.63(10)	0 (5)
AV♀×AV♂	0 (5)	0 (5)	85.02±6.57 (12)
AC♀×AF♂	70.80±12.00 (10)	57.88±15.14 (10)	\
AF♀×AC♂	\	\	\
AC♀×AV♂	6.85±6.15 (12)	\	62.44±7.77 (12)
AV♀×AC♂	15.53±19.10 (7)	\	63.97±11.38 (4)
AF♀×AV♂	\	\	\
AV♀×AF♂	\	60.00±15.81 (15)^1^	85.46±12.48 (12)^1^

The numbers in brackets are sample size.

1 The data were picked from [10].

For the combines of AF♀×AC♂ and AF♀×AV♂, too few neonate larvae to carry on survival experiments.

### Bacterial endosymbiont

We screened the totally 127 beetles for infection with *Wolbachia* using a PCR-based approach. Total genomic DNA of *Wolbachia* was extracted from whole beetles. The procedure of DNA extraction, amplification, and sequencing for *Wolbachia* is same to that for beetles described previously.

*Wolbachia* surface protein gene (*wsp*) was amplified with *Wolbachia*-specific primers (Table 3) to determine if *Wolbachia* were present. In order to check that the DNA extractions had been successful, we amplified the ITS2 region of beetles for each individual. Samples that appeared to be negative for *Wolbachia* were screened twice more.

The *wsp* of each individual were sequenced for strain identification. Because the *wsp* sequences were highly variable and fit for resolving the phylogenetic relationships of different *Wolbachia* strains [42], and there is very few variation of *wsp* sequences either among or within host species (see the results), we did not try to amplify and sequence other two common used but slower evolving genes, 16S rRNA gene and *ftsZ*, which were suggested can't provided sufficient information to adequately resolve the relationships between individual *Wolbachia* strains [42]–[44].

Standard PCR protocols were carried out using the following thermal profile: a pre-cycle denaturation at 94°C for 5 min, a post-cycle extension at 72°C for 10 min, and 35 cycles of a standard three-step PCR (94°C for 1 min, 55°C for 1 min, 72°C for 1 min). Successful PCR amplification and sequencing of target genes were confirmed by aligning the resulting sequences to *Wolbachia*. The three haplotypes are deposited in GenBank under the accession numbers JN903039–JN903041.

Few variations of *wsp* gene were detected, therefore, only the distribution of *wsp* haplotypes among beetles was summarized using statistical parsimony networks inferred in TCS 1.21 [45].

## Results

### Gene trees estimation

Gene genealogies were highly concordant for each dataset estimated by the ML, MP and Bayesian analyses, with variable bootstrap values (for MP, ML) or posterior probabilities (for MCMC). Phylogenetic analyses based on COI or COI/COII datasets suggested two clades, AF and AC+AV, but the lineage sorting of AC and AV is incomplete (Fig. 1a and Fig. 1c respectively); the monophyly of AF was well supported but with incomplete lineage sorting of AC and AV using COII dataset (Fig. 1b). Three clear clades (AC, AF and AV) and the relationship of AC+(AF+AV) were supported (Fig. 1d) using the intron of EF1α dataset, whereas, the monophyly of AC+AF was well supported when exon of EF1α dataset was used (Fig. 1e). The relationship among AC, AF and AV was unsettled, although the monophyly of these three clades was supported, based on EF1α (exon+intron) or ITS2 data (Fig. 1f and Fig. 1g respectively). The Incongruence Length Difference (ILD) tests indicated no significant incongruence among these partitions (*P = 0.087*), therefore, the concatenated data (five partitions) were fit for analysis together. Both three clear clades consisting with three tentative species and the relationship of AF+(AC+AV) were supported based on concatenated data (Fig. 1h).

### Species trees and divergence time estimation

**Figure 2:**
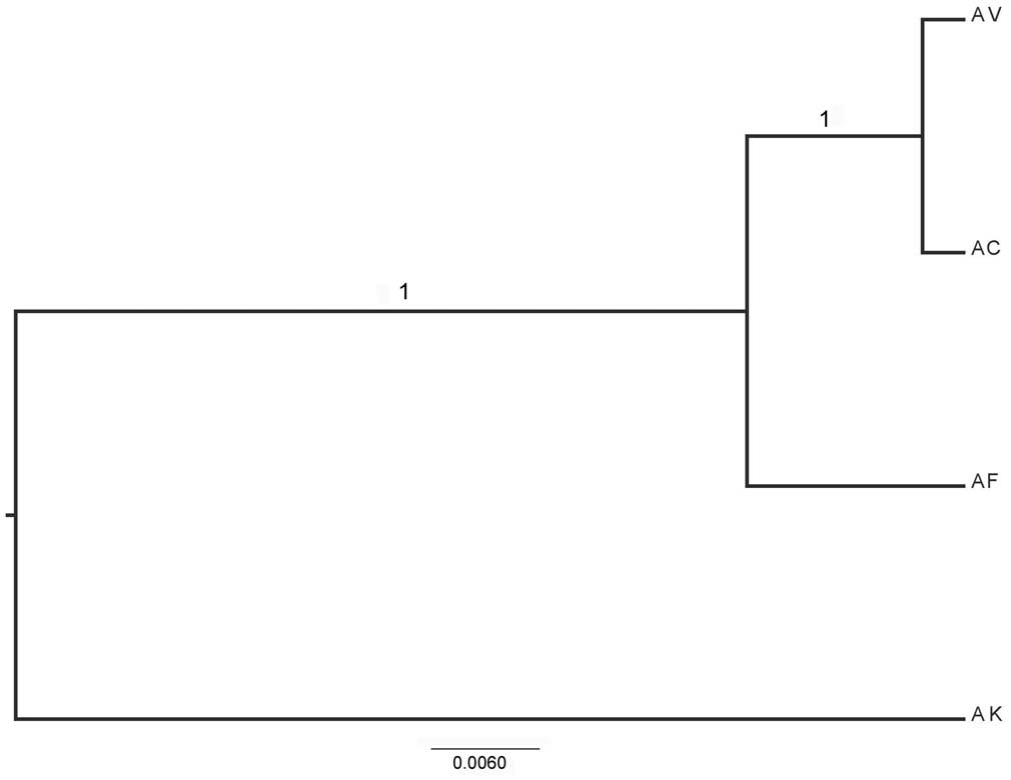
The estimate of the species tree based on the combined dataset.

**Figure 3:**
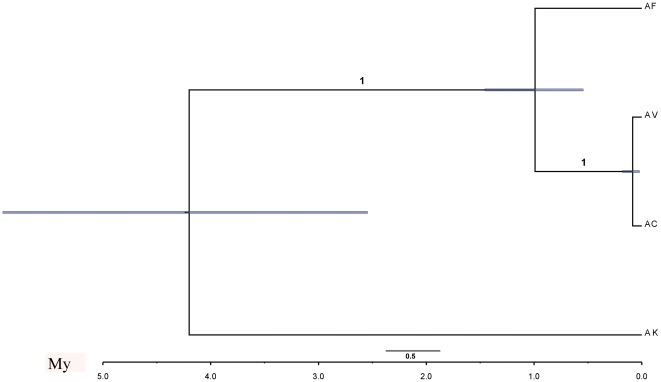
Phylogenetic tree based on COI and COII dataset with average pairwise divergence rates as 1.73% My^−1^ for COI and 1.38% My^−1^ for COII, constructed using *BEAST.

*BEAST analysis strongly support the relationships AF+(AC+AV) (Fig. 2). Based on the average pairwise substitution rates of COI and COII, the analysis showed that AF separated with AC+AV within 0.94 million years (95% CI, 0.52–1.40 My), and the divergence time between AC and AV is only 0.08 million years (95% CI, 0.02–0.16 My) (Fig. 3).

### Post-mating reproductive isolation

The hatch rates are always high (about 90%) for the intra-specific crosses. For AC-AF and AF-AV combinations, successful hybridisation occurred asymmetrically: the hatch rate is much lower when AF was the female parent (only 6.44% and 1.19% respectively) than when AF was the male parent (49.12% and 65.79% respectively); whereas, for AC-AV combination, the hatch rate is considerable in both directions (28.86% and 42.15% respectively, see Table 4).

The neonates of these three species only develop on their own host plants with high survival rates (about 85%–90%). For AC♀×AF♂ and AV♀×AF♂, the survival rates are comparatively high (about 65%–85%) on both maternal and paternal host plants. However, the survival rate of F1 neonates from both directions of AC-AV combination is much higher on *Geranium nepalens* than that on *Cirsium setosum* (62.44% vs. 6.85% and 63.97% vs. 15.53% respectively) (Table 5).

### Bacterial endosymbiont

In all three beetle species, almost 100% (except one individual of AF) of the individuals were found to be infected by *Wolbachia* (127 individuals totally; AC, n = 23; AF, n = 47; AV, n = 57) belong to “A” supergroup [42] (the result was not shown).

**Figure 4:**
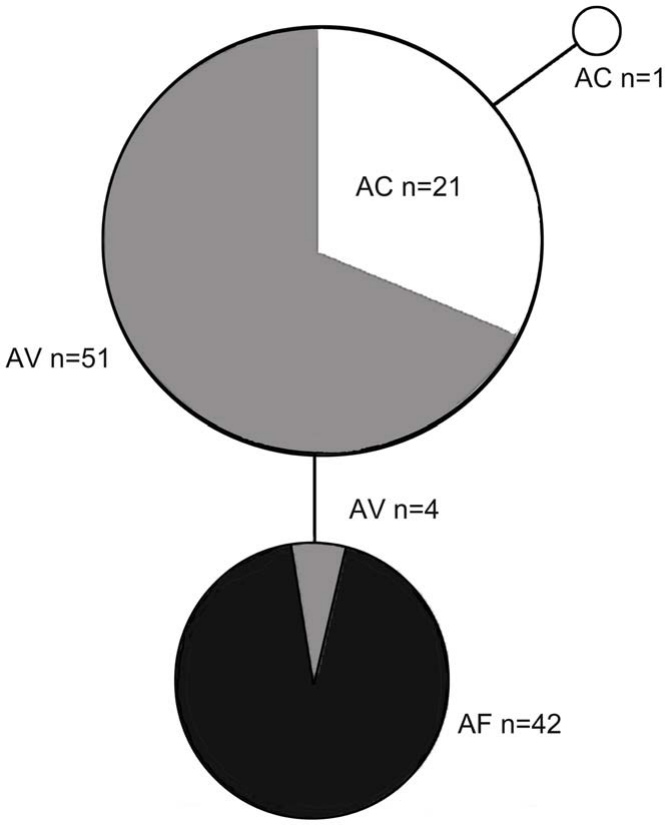
Statistical parsimony networks for *Wolbachia* surface protein gene (*wsp*) from three *Altica* species. Circles are proportional to the frequency of unique haplotypes. Only 3 haplotypes were detected in the totally 119 individuals (AC, n = 22; AF, n = 42; AV, n = 55). Among them, all of the AF individuals and four AV individuals belong to haplotype A; most individuals of AC and AV share the haplotype B; only one individual of AC belong to haplotype C.

Three *wsp* haplotypes were detected in the totally 119 individuals we sequenced successfully (AC, n = 22; AF, n = 42; AV, n = 55). Among them, all of the AF and four AV individuals belong to haplotype A; most individuals of AC and AV share the haplotype B; only one individual of AC belong to haplotype C (Fig. 4).

## Discussion

Insect species in nature are often incompletely isolated for millions of years after their formation [46], [47] and gene flow may persist even between non-sibling species [8], [48]–[50]. Therefore the viability of inter-specific offspring may in fact be common, and the incomplete post-mating reproductive isolation we document among these three beetles is not unexpected. The present study indicates that ITS2 and EF1α each show complete lineage sorting and provide useful tools for species identification, although they may not indicate the “true” relationship among species. Three clear clades in accordance with presumptive species were suggested based on concatenated sequence data. Furthermore, other multi-traits, for example, external and aedeagus morphological characters [51], feeding and oviposition preference [10], [14] and larval performance [10] confirmed these as full species.

Species level phylogenetic analysis allows insights into recent evolutionary history, especially patterns of divergence and speciation [52]. However, resolving phylogenies among closely related species can be extremely difficult when there is incongruence among gene trees [53], [54]. The use of a single gene sequence and one individual per taxon in a phylogenetic analysis is common but also potentially misleading [55]. It is therefore becoming popular to collect data sets containing multiple gene loci and multiple individuals per species [39]. Although there is incongruence among gene trees, the relationship of AF+(AC+AV) was well supported by both gene tree and species tree based on concatenated data.

Though molecular sequence data have become predominant in phylogenetic analyses, morphological, behavioral, and physiological traits are still also informative on phylogenetic relationships [56], [57]. In contrast to sexual isolation, post-mating isolation might evolve at a more regular rate, and many studies have reported a positive relationship between parental divergence and post-mating isolation [58], [59]. Here, unidirectional cytoplasmic incompatibility (CI) (see the egg hatching rates, table 4) was detected in AC-AF and AF-AV but not in AC-AV, and, may also suggest more close genetic affinity between AC and AV.

Almost 100% individuals of the three closely related flea beetles were found to be infected by *Wolbachia*. Then the prevalence of *Wolbachia* gave us an opportunity to infer relationship of the hosts based on sequence data of their endosymbiont. The haplotype network showed that most individuals of AC and AV share a same haplotype, may indicate their hosts' relationship as AF+(AC+AV), consistent with the result based on multi-locus sequence and post-mating reproductive data.

Therefore, our second hypothesis, “AV and AC are sister species which demonstrate incomplete post-mating isolation”, can't be rejected based on multi-locus sequence, post-mating reproductive and bacterial endosymbiont data. Pairwise incomplete post-mating isolation, relative short divergence time estimated by molecular data, and few variation of endosymbiont gene suggested recent speciation in these three sympatric specialists.

Many studies have showed that cytoplasmic incompatibility can be induced by the endosymbiont *Wolbachia* [17]–[20], [60]. To distinguish between *Wolbachia*-induced and nuclear-induced CI in this study system is significant to understand the essence and process of post-mating reproductive isolation and speciation. Present study showed that there is a few variations in the rapid evolution gene *wsp*, so it is doubtless that the *Wolbachia* in these three beetle species belong to same strain, and do not play a role in unidirectional CI [15], [17]–[20].

In any case, pairwise asymmetric post-mating isolations were detected, with different mechanisms. For AF♀×AC♂ and AF♀×AV♂ combinations were almost unable to produce viable offspring (Table 4), and suggests intrinsic hybrid inviability. On the other hand, for AC♀×AV♂, ecological hybrid inviability was detected. Although the eggs have a considerable hatch rate (28.86%, Table 4), the neonate larvae have a very low survival on maternal host plant (6.85%, Table 5), which the females of AC lay eggs on exclusively under conditions where they are given a choice (and even no-choice conditions, unpublished data). In this case, while high survival on their paternal host plant (62.44%) in laboratory is meaningless under field conditions, it does suggest the transmission and expression of genes required for use of this particular host. In addition, incomplete post-mating isolation (hatch rates) suggested potential introgression, however, considering almost complete lineage sorting (phylogenetic result), we speculated that effective pre-mating isolation among these three species should have evolved.

Several studies on closely related populations of phytophagous insects have shown that populations that have shifted to a novel host plant species have retained the ability to use their ancestral host [61]–[65], while some other specialists, for example, the swallowtail butterflies have lost the ability [66].These three flea beetles achieved distinct host specificity in a relative short time (0.94 My, see Fig. 3), that may be attributed to simple inheritance patterns of preference and performance [10], [14].

In some butterflies (*Papilio*), the host race formation is the result of, not the cause of genetic and ecological divergence [67], and the divergence achieved by temporal isolation but not feeding/habitat specialization [68]. The parental species of *Papilio* are also not isolated by pre-mating mate-preference barriers [69] nor by post-mating egg viability. In other Lepidoptera, there are combinations of reproductive isolating factors other than reduced egg viability. For examples, sexual selection by pheromones in European corn borers, visual mimicry and wing color mate preferences in *Heliconius* butterflies, local selection by natural enemies, as well as allochronic isolation [70]. While host specialization can play a very important role in divergence and even hybrid speciation of the fruit fly, *Rhagoletis* (Tephritidae) species [71]–[72], and other factors are generally needed for divergence and speciation [70]. However, in some other cases, for pea aphids and ladybird beetles, distinct host specificity alone can act as an almost complete pre-mating isolating barrier among insect populations, resulting in reproductive isolation in the absence of any post-mating barriers [73]–[78]. For our study system, we suggest that the unusual pattern of shifting between unrelated host plants and distinct host specificity should reduce the gene flow between host specific populations effectively and accelerate the speciation process of this species rich genus. To estimate the contribution of host plant to reproductive isolation and possible ecological speciation, further investigation is required to detect the potential gene flow among sympatric populations of these beetles and to understand how the effective pre-mating reproductive isolating mechanisms have evolved.
